# The complete chloroplast genome sequence of the relict woody plant *Metasequoia glyptostroboides* Hu et Cheng

**DOI:** 10.3389/fpls.2015.00447

**Published:** 2015-06-16

**Authors:** Jinhui Chen, Zhaodong Hao, Haibin Xu, Liming Yang, Guangxin Liu, Yu Sheng, Chen Zheng, Weiwei Zheng, Tielong Cheng, Jisen Shi

**Affiliations:** ^1^Key Laboratory of Forest Genetics and Biotechnology, Ministry of Education, Nanjing Forestry UniversityNanjing, China; ^2^College of Biology and the Environment, Nanjing Forestry UniversityNanjing, China; ^3^School of Life Sciences, Huaiyin Normal UniversityHuaian, China

**Keywords:** relict plant, *Metasequoia glyptostroboides*, chloroplast genome, inverted repeat, cupressophytes, conifer evolution

## Abstract

*Metasequoia glyptostroboides* Hu et Cheng is the only species in the genus *Metasequoia* Miki ex Hu et Cheng, which belongs to the Cupressaceae family. There were around 10 species in the *Metasequoia* genus, which were widely spread across the Northern Hemisphere during the Cretaceous of the Mesozoic and in the Cenozoic. *M. glyptostroboides* is the only remaining representative of this genus. Here, we report the complete chloroplast (cp) genome sequence and the cp genomic features of *M. glyptostroboides*. The *M. glyptostroboides* cp genome is 131,887 bp in length, with a total of 117 genes comprised of 82 protein-coding genes, 31 tRNA genes and four rRNA genes. In this genome, 11 forward repeats, nine palindromic repeats, and 15 tandem repeats were detected. A total of 188 perfect microsatellites were detected through simple sequence repeat (SSR) analysis and these were distributed unevenly within the cp genome. Comparison of the cp genome structure and gene order to those of several other land plants indicated that a copy of the inverted repeat (IR) region, which was found to be IR region A (IRA), was lost in the *M. glyptostroboides* cp genome. The five most divergent and five most conserved genes were determined and further phylogenetic analysis was performed among plant species, especially for related species in conifers. Finally, phylogenetic analysis demonstrated that *M. glyptostroboides* is a sister species to *Cryptomeria japonica* (L. F.) D. Don and to *Taiwania cryptomerioides* Hayata. The complete cp genome sequence information of *M. glyptostroboides* will be great helpful for further investigations of this endemic relict woody plant and for in-depth understanding of the evolutionary history of the coniferous cp genomes, especially for the position of *M. glyptostroboides* in plant systematics and evolution.

## Introduction

*Metasequoia glyptostroboides* Hu et Cheng, also known as the dawn redwood or ShuiShan in Chinese (endemic to China), is a well-known “living fossil" tree in plant taxonomy. There were around 10 species in the *Metasequoia* genus, which were once widely distributed across the Northern Hemisphere during the Cretaceous of the Mesozoic and in the Cenozoic. *M. glyptostroboides* is the only relict of this genus and was re-discovered in the early 1940s and denominated by Professors Hu and Cheng (Hu and Cheng, 1948; Chaney, 1950; Hu, 1980; Yang, 1999). The distribution of remaining dark wood and stumps (diameter size in 2–4 m) covered by alluvial deposits, and the relic living trees of *M. glyptostroboides* are restricted to an extremely enclosed valley that is 30 km (south–north) by 20 km (east–west), along with the jointed boundary of the Sichuan, Hubei, and Hunan provinces in central China (Hu and Cheng, 1948; Bartholomew et al., 1983), even though it has been introduced and then planted worldwide after the 1940s. Since it was first described in 1948 (Hu and Cheng, 1948), *M. glyptostroboides* has been the focus of much attention by plant scientists worldwide. It has its own conservation organization called the “Save the Dawn Redwoods League," and it has been listed as a critically endangered species (in the Red List) by the International Union for Conservation of Nature (Walter and Gillett, 1998).

Chloroplasts, one member of a family of organelles and the key place for the photosynthetic processing in plants, have been assessed for their roles in plant physiology and biochemistry (Neuhaus and Emes, 2000; Dyall et al., 2004). In the genomic and post-genomic era, the mining of genomic information of plant organelles, especially the plant cp genome, is now also of interest. Approximately, 644 plastid genomes in Viridiplantae have been sequenced and deposited in the NCBI Organelle Genome Resources^1^, since the first reports of complete cp genome sequences from tobacco (Shinozaki et al., 1986) and liverwort (Ohyama et al., 1986) in 1986. Interestingly, most of these sequenced cp genomes have a typical quadripartite structure with a pair of inverted repeats (IRs) separated by a large single-copy region (LSC) and a small single-copy region (SSC), and they ranged from 120 to 160 Kb in length (Sugiura, 1992). The pair of IRs, a prominent feature of most land plant cp genomes, varied from 6 to 76 Kb in length (Palmer, 1985) and some reports proposed that the cp genome size might be influenced by the length of the pair of IRs (Chang et al., 2006; Wang et al., 2008; Guisinger et al., 2011). Palmer and Thompson (1981) found that one of the IRs has been lost in some legume plants, and they concluded that rearrangements that change the homologous gene order within the cp genome occurred at a higher frequency during legume evolution. Species in the Ginkgoales (Lin et al., 2012), Cycadales (Wu et al., 2007), and Gnetales (Wu et al., 2009) orders have retained both IRs, which ranged from 17.3 to 25.1 Kb. In contrast to these gymnosperms, a wide taxonomic range of coniferous cp genomes lack one copy of the IRs. Strauss et al. (1988) concluded that the cp genomes of conifers have a higher rearrangement frequency than those of most higher plants. Furthermore, Wu et al. (2011) also found that there were two independent losses of an IR copy in conifer evolution. Wu and Chaw (2014) further reinforced this proposition by showing that an IRB has been lost from cp genomes of Pinaceae and IRA has been lost from those of the Cupressophytes.

These complete cp genome sequences have been widely used in developing useful molecular markers (Kung, 1989; Awasthi et al., 2012; Dong et al., 2012; Jheng et al., 2012; Chen and Melis, 2013) and for molecular phylogenetic studies (Cai et al., 2006; Jansen et al., 2006, 2007; Samson et al., 2007; Hirao et al., 2008; Tangphatsornruang et al., 2009; Takano and Okada, 2011; Turner et al., 2013; Gaudeul et al., 2014). Here, we report the first complete genome sequence of *M. glyptostroboides* (GenBank accession number: KR061358) based on Illumina high-throughput sequencing technology. Thus, the complete cp genome sequence of *M. glyptostroboides*, in conjunction with previously published cp genome sequences, will help to expand our understanding of the evolutionary history of the coniferous cp genomes, especially with respect to positioning *M. glyptostroboides* in plant systematics and evolution.

## Materials and Methods

### DNA Sequencing and Genome Assembly

Using the high salt concentrations method introduced by Sandbrink et al. (1989), 5 μg of cp DNA was isolated from 20 g of fresh young leaves of *M. glyptostroboides* grown at Nanjing Forestry University for 3 days in the dark. From the DNA sample, a 500-bp paired-end library was constructed using the cp DNA, and ∼2 GB of sequence, with an average read length of 301 bp, was obtained on the MiSeq platform.

The complete cp genome sequence was assembled as follows. First, the MiSeq reads was trimmed to 200 bp in length using an in-house ‘fasta_length_trimmer’ script to remove the potential low quality bases. Then, the initial contigs were produced with cleaned reads using Velvet Assembler version 1.2.07 (Zerbino and Birney, 2008). Contigs were selected for assembly if they showed similarity to the published cp genome. Finally, we got one single circular cp genome sequence (131,887 bp) without ambiguous (N) bases by linking these contigs with paired-end MiSeq reads using SSPACE premium version 2.2 (Boetzer et al., 2011) followed by a manual check.

### Genome Annotation and Sequence Statistics

The cp genome of *M. glyptostroboides* was annotated through the online program Dual Organellar GenoMe Annotator (DOGMA; Wyman et al., 2004). DOGMA annotations were manually checked, and the start and stop codons adjusted by comparison to homologous genes from other sequenced cp genomes. In addition, all transfer RNA genes were verified by using tRNAscan-SE version 1.21 (Schattner et al., 2005) with default settings. The circular *M. glyptostroboides* cp genome map was drawn using the OGDRAW program (Lohse et al., 2007). Codon usage and GC content were analyzed by MEGA5 (Tamura et al., 2011).

### Repeat Structure and Sequence Analysis

Tandem Repeats Finder version 4.07b (Benson, 1999) was used to identify tandem repeats in the *M. glyptostroboides* cp genome with default settings. Forward repeats and palindromic repeats were identified by using REPuter (Kurtz et al., 2001) with a minimal size of 30 bp and >90% identity (Hamming distance equal to 3) between the two repeat copies. Simple sequence repeats (SSRs) were detected using the Perl script MISA^2^ with a motif size of one to six nucleotides and thresholds of eight, four, four, three, three, and three repeat units for mono-, di-, tri-, tetra-, penta-, and hexanucleotide SSRs, respectively. All of the repeats identified with the various programs were manually verified to remove redundant results.

### Sequence Divergence and Phylogenetic Analysis

We used 82 protein-coding genes to calculate the average pairwise sequence divergence among the cp genomes from 28 coniferous species. These cp genomes came from six families in the Pinales lineage of gymnosperms: Araucariaceae, Cephalotaxaceae, Cupressaceae, Pinaceae, Podocarpaceae, and Taxaceae. The missing and abnormal gene annotations were re-annotated in some taxa after comparison of cp gene order and multiple sequence alignments during the comparative sequence analysis. The orthologous genes were aligned using the Align Codon option of ClustalW (Thompson et al., 1994) implemented in MEGA5 (Tamura et al., 2011). Each average pairwise sequence divergence was calculated using Kimura’s two-parameter model (Kimura, 1980).

For phylogenetic analysis, we selected 30 cp genomes (Supplementary Table S5), of which 28 were from species representing the six families within the Pinales and two were from species *Ginkgo biloba* and *Cycas revoluta*, set as the outgroups. For each cp genome, we previously extracted 64 protein-coding genes that were present in all of these cp genomes. All of these orthologous genes were individually aligned with ClustalW (Thompson et al., 1994) and with manual adjustment. Then we used the entropy-based index of substitution saturation (Xia et al., 2003) implemented in DAMBE (Xia and Lemey, 2009). Orthologous genes with Iss ≥Iss.c (the critical Iss value), indicating that they had experienced severe substitution saturation, were excluded (Supplementary Table S7), ultimately resulting in 47 orthologous genes. For each orthologous gene, the alignment was trimmed by using trimAL version 1.2 (Capella-Gutiérrez et al., 2009), and the trimmed alignments were concatenated by SequenceMatrix version 1.7.8 (Vaidya et al., 2011). Finally, a concatenated 47-gene nucleotide sequence matrix of 34,710 bp was produced.

Maximum parsimony (MP) and maximum likelihood (ML) analyses based on the 47 protein-coding genes of the 30 species were performed. For MP analysis, PAUP^∗^4.0b10 (Swofford, 2003) was implemented using a heuristic search with 1000 random taxon addition replicates and tree-bisection-reconnection branch swapping with the Multrees option. We performed 1000 non-parametric bootstrap replicates with tree-bisection-reconnection branch swapping to assess uncertainty in the MP topology. For ML analysis, phylogenetic trees were constructed with the custom model in PhyML 3.0 (Guindon et al., 2010). The custom option was used to implement a General Time Reversible + Proportion Invariant + Gamma (GTR+I+G) nucleotide substitution model, which was selected to be the best-fit model by Modeltest 3.7 (Posada and Crandall, 1998). To estimate tree topologies, subtree pruning, and regrafting were performed on five random BioNJ calculated starting trees. Also, for bootstrap analysis, we performed 1000 non-parametric bootstrap replicates to estimate the support of the data for each internal branch of the phylogeny.

## Results and Discussion

### Genome Size and Content

**FIGURE 1:**
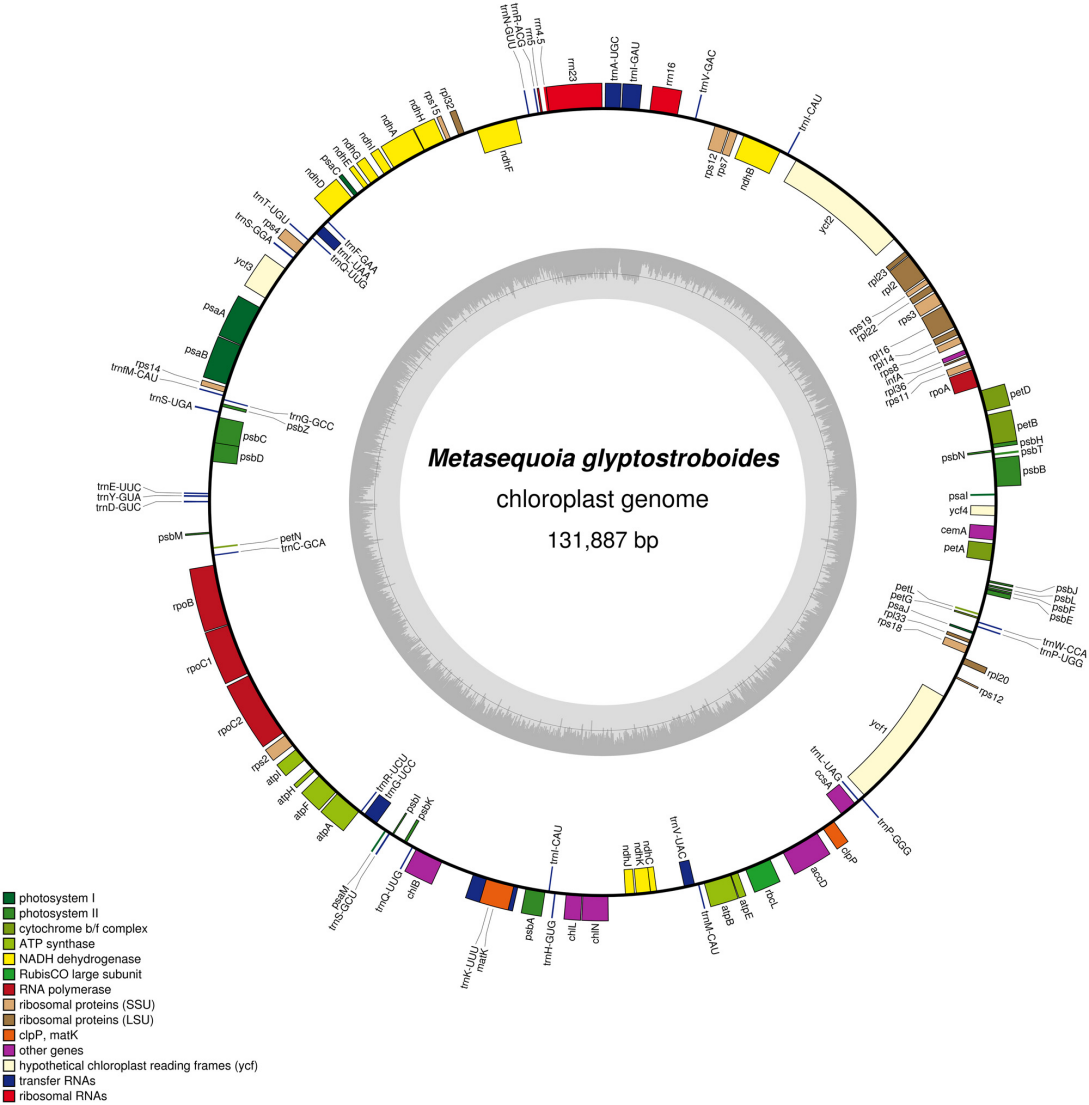
**Gene map of the *Metasequoia glyptostroboides* cp genome**. Genes shown inside of the circle are transcribed clockwise, whereas genes transcribed counter-clockwise are depicted on the outside. Genes belonging to different functional groups are color-coded. GC content is represented on the inner circle by dark gray bars, and AT content is represented by lighter gray bars.

The size of the *M. glyptostroboides* cp genome was found to be 131,887 bp (**Figure 1**), similar to those of other sequenced cp genomes from Cupressophytes, which range from 127 to 146 Kb (Guo et al., 2014; Wu and Chaw, 2014). Notably, the cp DNA of *M. glyptostroboides* is circular, without the typical quadripartite structure that contains a pair of IRs separated by LSC and SSC regions (**Figure 1**). However, there were inverted regions containing duplicated *trnI-CAU* genes in the *M. glyptostroboides* cp genome. Such regions were presumed to be residues of typical IRs in *Cryptomeria japonica* D. Don (Hirao et al., 2008), *Agathis dammara*, and *Calocedrus formosana* (Wu and Chaw, 2014). The overall AT content of the *M. glyptostroboides* cp genome was 64.7% (**Table 1**), which is higher than that of *A. dammara* (63.46%; Wu and Chaw, 2014) and *Nageia nagi* (62.74%; Wu and Chaw, 2014); similar to that of *C. japonica* (64.62%; Hirao et al., 2008); and lower than that of *Cephalotaxus wilsoniana* (64.92%; Wu et al., 2011), *C. formosana* (65.17%; Wu and Chaw, 2014), and *Taiwania cryptomerioides* (65.37%; Wu et al., 2011).

**Table 1:** Chloroplast genome features in *Metasequoia glyptostroboides*.

	T/U (%)	C (%)	A (%)	G (%)	Length (bp)	GC (%)
Genome	32.5	17.2	32.2	18.1	131,887	35.3
tRNA genes	24.6	24.1	22.2	29.1	2,327	53.2
rRNA genes	30.1	22.4	26.9	30.6	4,587	53.0
Protein-coding genes	31.6	16.9	31.6	19.9	74,088	36.8
Firstst position	23.0	18.3	30.6	27.7	24,696	46.0
Second position	33.0	20.1	30.4	16.8	24,696	36.9
Third position	39.0	12.3	33.9	15.1	24,696	27.4

A total of 117 genes were identified in the *M. glyptostroboides* cp genome, of which 115 are unique and 2, *trnI-CAU* and *trnQ-UUG*, are duplicated (Supplementary Table S1). Among the 115 unique genes, 15 contain one intron (nine protein-coding and six tRNA genes) and 2 (*rps12* and *ycf3*) contain two introns (**Table 2**; Supplementary Table S1). *Rps12* is a trans-spliced gene (Hildebrand et al., 1988) with N-terminal exon I being 95 Kb pairs downstream of the C-terminal exons II and III. The *trnK-UUU* gene has the largest intron (2439 bp), which includes the *matK* gene. In addition, two genes, *rps16* and *trnT-GGU*, were identified as pseudo-genes.

**Table 2:** The genes in the *M. glyptostroboides* cp genome with introns.

Gene	Start	End	Exon	I (bp)	Intron	I (bp)	Exon	II (bp)	Intron	II (bp)	Exon	III (bp)
*atpF*	82,041	83,302	159	692	411		
*ndhA*	42,838	44,711	558	767	549		
*ndhB*	25,280	23,114	723	688	756		
*petB*	2,981	4,673	6	1,045	642		
*petD*	4,867	6,014	8	650	490		
*rpl16*	10,989	9,682	9	888	411		
*rpl2*	14,216	12,737	398	649	433		
*rpoC1*	72,590	75,377	433	700	1,655		
*rps12*^∗^	122,289	26,111	114	-	232	534	26
*rps18*	124037	123588	27	75	348		
*trnA-UGC*	32,032	32,861	38	757	35		
*trnG-UCC*	86,042	85,206	24	764	49		
*trnI-GAU*	31,001	31,979	42	902	35		
*trnK-UUU*	91,877	94,370	30	2,439	25		
*trnL-UAA*	50,162	49,584	35	494	50		
*trnV-UAC*	103,885	103,288	39	522	37		
*ycf3*	52,642	54,554	126	699	228	704	156

*Rps12 is a trans-spliced gene.

**FIGURE 2:**
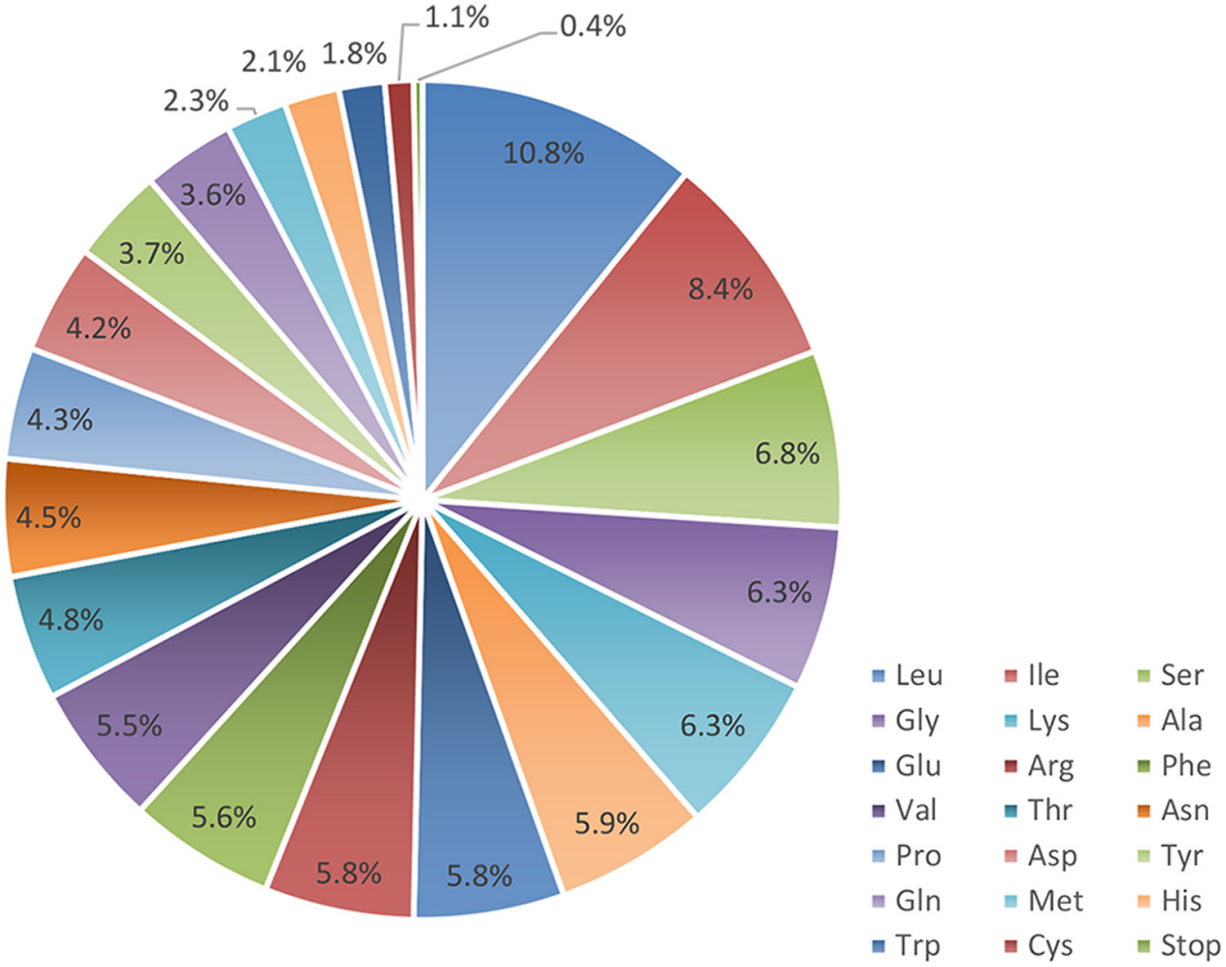
**Amino acid frequencies in the *M. glyptostroboides* cp protein-coding genes**. The frequencies of amino acids were calculated for all of the 82 protein-coding genes from the start codon to the stop codon in the *M. glyptostroboides* cp genome excluding introns.

Proteins, tRNAs and rRNAs are encoded by 56.18, 1.76, and 3.48% of the genome sequence, respectively, (**Table 1**). The remaining 38.58% of the genome is non-coding regions, including intergenic spacers, introns and pseudo-genes. Protein-coding regions are 74,088 bp in length and contain 82 protein-coding genes coding for 24,696 codons. The frequency of codon usage was deduced for the *M. glyptostroboides* cp genome based on the sequences of protein-coding genes and tRNAs. Notably, leucine (10.8%) and cysteine (1.1%) were the most-frequently and least-frequently coded amino acids, respectively, (**Figure 2**). Among codons, the most and least used were AAA (1171), encoding lysine, and UGC (68), encoding cysteine, respectively, (Supplementary Table S2). Furthermore, the AT content was 54.0, 63.1, and 72.6% at the first, second, and third codon positions, respectively, within protein-coding regions (**Table 1**). The bias toward a higher AT content at the third codon position is consistent with the enrichment of A and T that has been widely observed in many other sequenced land plant cp genomes (Morton, 1998; Tangphatsornruang et al., 2009; Nie et al., 2012; Qian et al., 2013).

### Repeat and SSR Analysis

**FIGURE 3:**
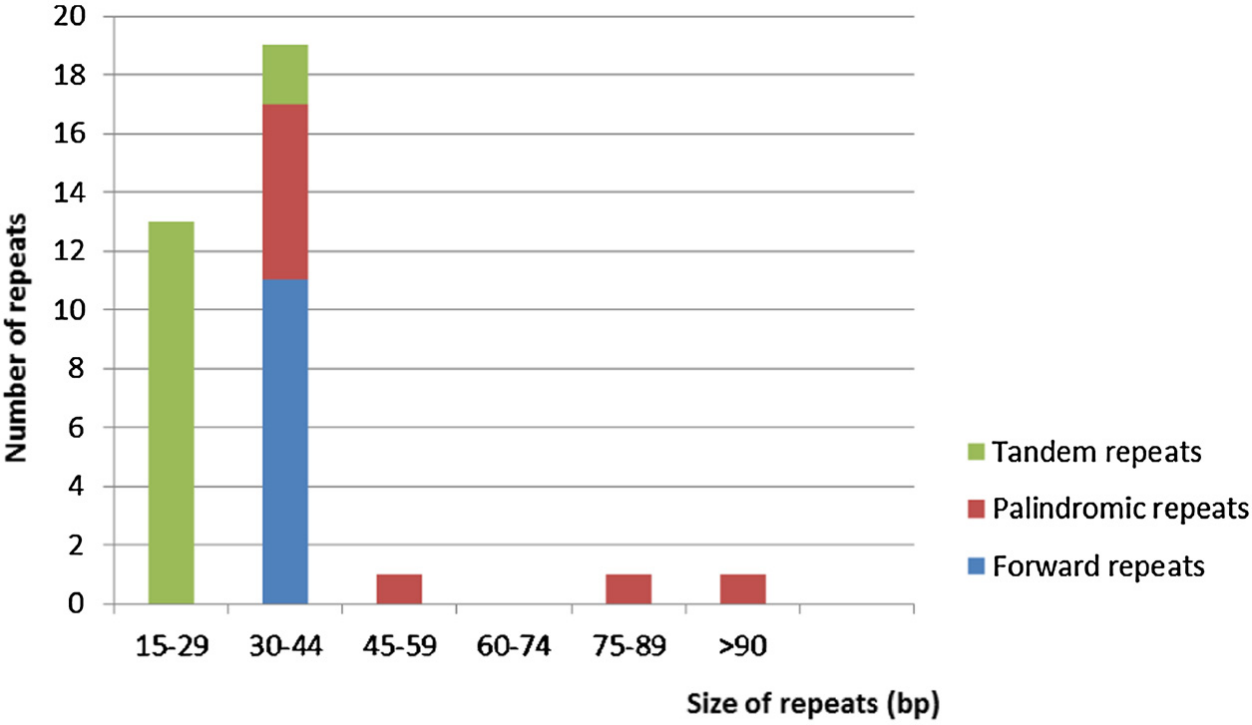
**Frequency of repeats by length in the *M. glyptostroboides* cp protein-coding genes**. The cutoff value for tandem repeats was 15 bp and for forward repeat and palindromic repeat was 30 bp.

Repeat motifs are very useful in the analysis of genome rearrangement and also play an important role in phylogenetic analysis (Cavalier-Smith, 2002; Nie et al., 2012). Analysis of the *Cephalotaxus oliveri* cp genome further supports that repeats are crucial in inducing substitutions and indels (Yi et al., 2013). For repeat analysis, 11 forward repeats, nine palindromic repeats, and 15 tandem repeats were detected in the *M. glyptostroboides* cp genome (Supplementary Table S3). Among these repeats, all of the forward repeats were 30–44 bp in size, whereas only two tandem repeats were 30–44 bp in length, and the rest were 15–29 bp (**Figure 3**). At the same time, six palindromic repeats were 30–44 bp, and three other palindromic repeats were 48, 86, and 278 bp. Overall, 35 repeats were detected in the *M. glyptostroboides* cp genome. Most of these repeats (40.0%) were distributed in the intergenic spacer regions, whereas 25.7% were in the protein-coding regions, and 5.7% were in the introns. In addition, four pairs of repeats were associated with tRNA genes and one forward repeat was located in rrn23. The remaining four pairs of repeats were distributed in two different regions, the intergenic spacer regions and the introns or protein-coding regions. These repeat motifs were selected for population studies and phylogenetic analysis because they are an informative source for developing markers (Nie et al., 2012).

**FIGURE 4:**
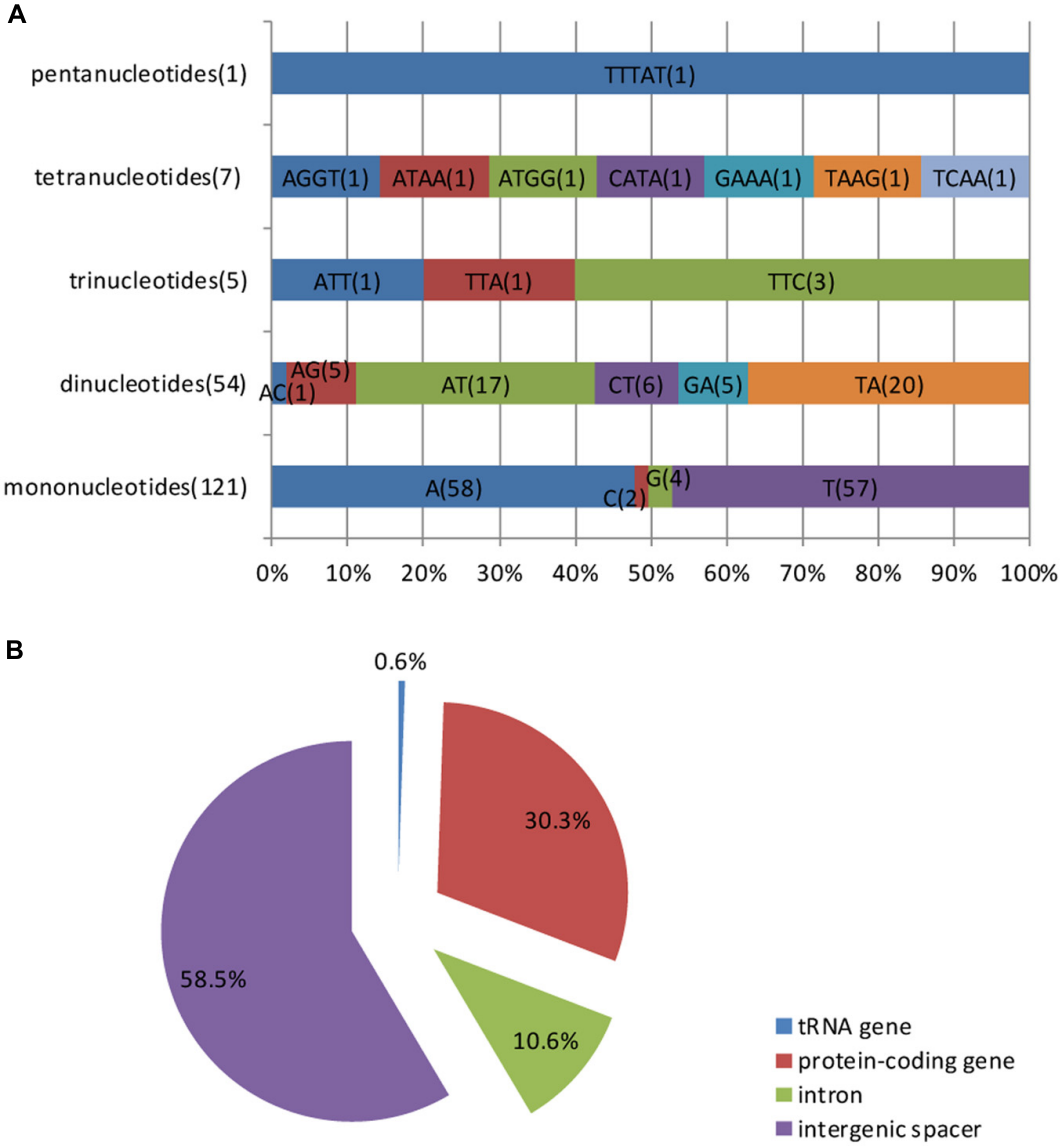
**Simple sequence repeat (SSR) analysis in the *M. glyptostroboides* cp genome. (A)** Frequency of identified SSR motifs in different repeat type classes. **(B)** Location distribution of all of the SSR motifs.

Simple sequence repeats, also known as microsatellites or short tandem repeats, are repeating sequences of 1–6 bp and are widely distributed over the genome. Microsatellites are typically co-dominant and have a higher degree of polymorphism (Weber, 1990). Because of these characteristics, SSRs are excellent molecular markers (Gupta et al., 1996) and are widely used in molecular marker assisted breeding (Rafalski and Tingey, 1993), population genetics (Powell et al., 1995), genetic linkage map construction and gene mapping (Pugh et al., 2004). Based on the SSR analysis, a total of 188 perfect microsatellites were detected in the *M. glyptostroboides* cp genome, of which 121 are mononucleotides, 54 are dinucleotides, five are trinucleotides, seven are tetranucleotides, and one is a pentanucleotide (Supplementary Table S4). Among these SSRs, most of the mononucleotides (95.04%) are composed of A/T and the majority of dinucleotides (68.52%) are composed of AT/TA, whereas the rest of the SSRs have a high A/T content (**Figure 4A**). These results are consistent with the contention that cp SSRs are generally composed of short polyadenine (polyA) or polythymine (polyT) repeats (Kuang et al., 2011). The higher A/T content in cp SSRs also contributes to a bias in base composition, such that A/T is enriched (64.7%) in the *M. glyptostroboides* cp genome.

**Figure 4B** shows the SSR distribution in the *M. glyptostroboides* cp genome. It was clearly indicated in **Figure 4B** that SSRs are more abundant in the non-coding than protein-coding regions, which account for 58.5 and 30.3% of all SSRs detected, respectively. In addition, the 10.6% of SSRs in the introns and 0.6% in tRNA genes. Moreover, although protein-coding genes account for 56.2% of the total cp genome length, only 30.3% of the SSRs exist within these regions, which suggests an uneven distribution of SSRs within the *M. glyptostroboides* cp genome.

### Loss of IRA

**FIGURE 5:**
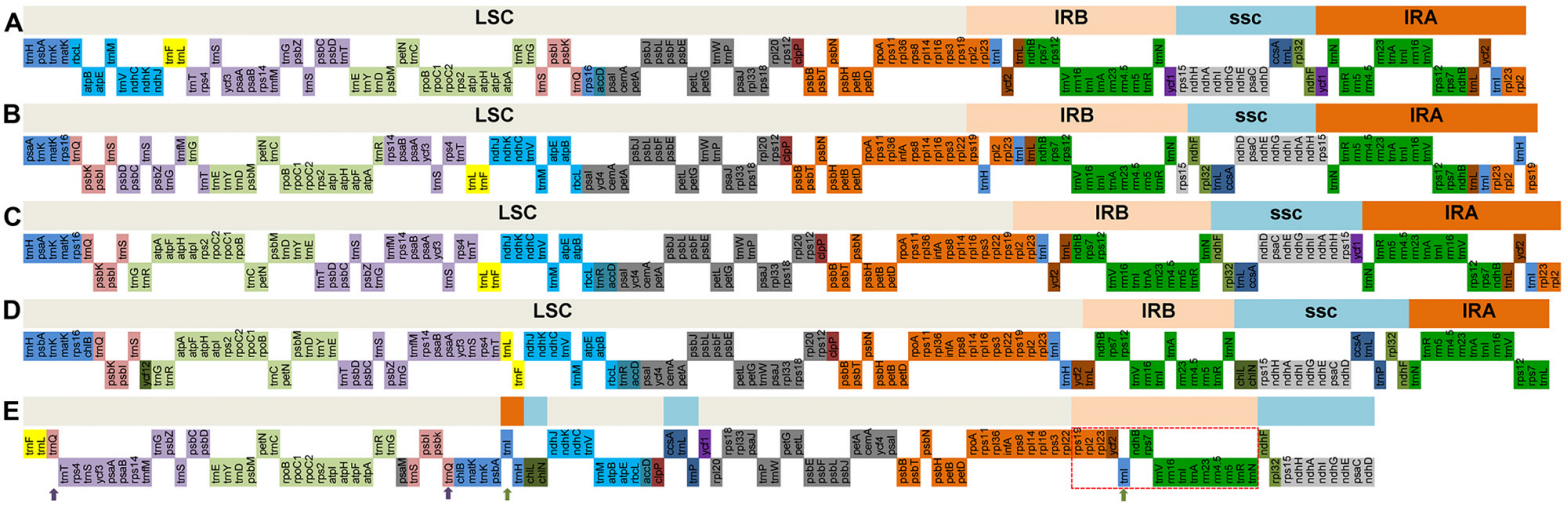
**Comparisons of cp genomic structure among five land plants, including *M. glyptostroboides***. Each colored gene segment has the same gene order in the five land plants. Each colored box for each gene order represents different regions [large single-copy (LSC), IRB, SSC, and IR region A (IRA)] in *Glycine max* **(A)**, *Oryza australiensis* **(B)**, *Nymphaea alba* **(C),** and *Ginkgo biloba* **(D)**. In *M. glyptostroboides* **(E)**, the purple and cyan arrows show two duplicated genes, which are *trnQ-UUG* and *trnI-CAU*, respectively, and the pink dashed box is believed to correspond to the IRB, which is retained in this species.

Interestingly, we noticed that the *M. glyptostroboides* cp genome does not have a typical quadripartite structure (**Figure 1**). We thus compared the cp genomic structure of *M. glyptostroboides* (representing Cupressophytes) with that from four other land plant species, *Glycine max* (representing dicots), *Oryza australiensis* (representing monocots), *Nymphaea alba* (representing basal angiosperm), and *G. biloba* (representing Ginkgo). As shown in **Figure 5E**, the region of the *M. glyptostroboides* cp genome corresponding to the SSC of *G. biloba* was divided into three segments, and the relevant LSC region was divided into three segments. One of the IRs that is present in the other land plants was lost in the *M. glyptostroboides* cp genome. The 495-bp IR in the *Pinus thunbergii* cp genome, which contains a duplicated *trnI-CAU* gene and a partial *psbA* gene, and the 114-bp IR in the *C. japonica* cp genome, which contains a duplicated *trnI-CAU* gene, were both presumed to be the residual IR (Tsudzuki et al., 1992; Hirao et al., 2008). Also, there are two IRs in the *M. glyptostroboides* cp genome (Supplementary Table S4), a 278-bp IR containing a duplicated *trnQ-UUG* gene (purple arrows in **Figure 5E**) and an 86-bp IR containing a duplicated *trnI-CAU* gene (cyan arrows in **Figure 5E**). Hirao et al. (2008) reported that the *C. japonica* cp genome contains a duplicated *trnQ-UUG* gene, and they postulated an inversion event occurred in the region from *trnQ-UUG* to *trnT-UGU* to explain this phenomenon. Based on these analyses, we believe that the 86-bp IR containing the duplicated *trnI-CAU* gene is the residual IR in the *M. glyptostroboides* cp genome.

As a result of the loss of one large IR copy from the *M. glyptostroboides* cp genome, a gene segment containing the entire *ycf2* gene and the rRNA operon (the pink dashed box in **Figure 5E**) was trans-located downstream of the *psbB*–*rpl22* gene cluster. These structural features in the *M. glyptostroboides* cp genome were in accord with those of IRB in the other four land plant cp genomes (**Figures 5A–D**), namely that the IRA region is always upstream of *psbA*, whereas the IRB region is always downstream of the *psbB*–*rpl22* gene cluster (the cp genome of *G. max* has lost *rpl22*). Based on a previous report, the gene order of the LSC-IR junction is conserved, and the regions encompassing the entire *ycf2* gene and adjoining *psbA* or *rpl23*–*rps3* gene cluster should correspond to the retained ancestral IRs (Wu et al., 2011). We thus reasonably speculate that the IRA has been lost but the IRB has been retained in the *M. glyptostroboides* cp genome.

### Sequence Divergence of Protein-Coding Genes

We compared the cp gene content and calculated average pairwise sequence divergence of 82 protein-coding genes among 28 conifers (Supplementary Table S5), and the results are summarized in Supplementary Table S6. Of these genes, 52% had an average sequence divergence of >0.10, and the five most divergent genes were *ycf1* (0.60), *clpP* (0.46), *ycf2* (0.43), *infA* (0.33), and *accD* (0.31). Among these five genes, *ycf1* and *ycf2* have large sequence length variation, 3393 and 1281 bp, respectively. The length variation of *ycf1* is largely due to an indel mutation, and the length variation of *ycf2* is mostly caused by an internal indel mutation associated with direct repeat sequences (Kim and Lee, 2004). More interestingly, 74% of the genes that varied in length by <10 bp had relatively low evolutionary divergence (<0.10). In contrast, 73% of genes that had an average sequence divergence of >0.15 varied in length by >50 bp. These data suggested that genes conserved at the sequence level also have less length variation.

The *rps* class of genes showed relatively high evolutionary divergence, ranging from 0.09 to 0.19, similar to the *rpl* gene class (0.10–0.24), whereas the majority of genes in the *psa*, *psb*, and *pet* classes had relatively low evolutionary divergence (<0.10). The data suggested that the divergence levels of cp genes are associated with the functional constraints of the genes. Similar results were also observed in 30 asterid cp genomes (Qian et al., 2013) and 16 other vascular plant cp genomes (Kim and Lee, 2004).

If both the sequence divergence and length variation of genes are considered, we believe that *ycf1*, *accD*, *ycf2*, *clpP*, and *rpl32*, which have relatively high sequence divergence and length variation, are good candidates for phylogenetic study among closely related conifer species. In contrast, *psbN*, *psbF*, *ndhE*, *psbA*, and *psaC*, which have relatively low sequence divergence and length variation, are proper candidates for phylogenetic study of higher plants.

### Phylogenetic Analysis

To examine the phylogenetic position of *M. glyptostroboides* within the conifers, we previously selected 64 orthologous protein-coding genes that are commonly present in the cp genomes of 30 gymnosperm species. Among these species, 28 represent six families within the Pinales, including Araucariaceae, Cephalotaxaceae, Cupressaceae, Pinaceae, Podocarpaceae, and Taxaceae and two species, *G. biloba* and *C. revoluta*, were set as outgroups. We used an index reported by Xia et al. (2003) to measure the degree of substitution saturation, which has an effect on phylogenetic reconstruction (Dávalos and Perkins, 2008), and ultimately identified 47 orthologous protein-coding genes (Supplementary Table S7). After concatenating trimmed alignments, the 47-gene nucleotide sequence matrix of 34,710 bp was used to perform phylogenetic analysis.

**FIGURE 6:**
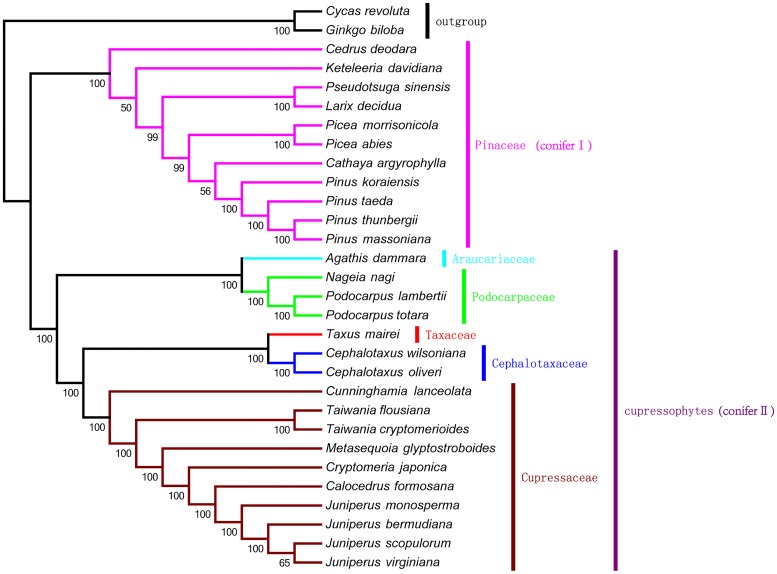
**The maximum parsimony (MP) phylogenetic tree based on 47 protein-coding genes**. The MP tree has a length of 21,985, with a consistency index of 0.6723, and a retention index of 0.8701. Numbers below each node are bootstrap support values. *G. biloba* and *Cycas revoluta* were set as outgroups.

We used MP and ML analysis to construct the evolutionary tree. With MP analysis we constructed a single tree with a length of 21,985, a consistency index of 0.6723, and a retention index of 0.8701 (**Figure 6**). Bootstrap analysis showed that there were 27 of 30 nodes with bootstrap values ≥99%, and most of these nodes had 100% bootstrap values. With ML analysis we also constructed a single tree with -lnL of 166,544.48908 by using the GTR+I+G nucleotide substitution model (Supplementary Figure S1). The bootstrap values were very high in the ML tree, with values of 100% for 28 of 30 nodes. The MP and ML trees had similar phylogenetic topologies, with two major clades: conifer I and conifer II. Conifer I included only the pine family (Pinaceae), whereas conifer II, namely Cupressophytes, included the remaining families (i.e., Araucariaceae, Cephalotaxaceae, Cupressaceae, Podocarpaceae, and Taxaceae). In conifer I, the bootstrap values for the sister relationship between *Cathaya argyrophylla* and the *Pinus* genus were low, 56% in the MP tree and 39% in the ML tree. In addition, *Pseudotsuga sinensis* was placed as a sister to *Larix decidua*, with 100% bootstrap values in both the MP and ML trees, which is consistent with the result of (Ran et al., 2010). Within conifer II, there were three major sub-clades comprised of Araucariaceae and Podocarpaceae, Taxaceae and Cephalotaxaceae, and Cupressaceae. This phylogenetic topology was congruent with the results of previous phylogenetic analysis of plastid genes from Cupressophytes (Guo et al., 2014). Both the MP and ML phylogenetic results strongly support that *M. glyptostroboides* is a sister species to *C. japonica* and to *T. cryptomerioides*.

## Conclusion

We present the complete cp genome sequence (131,887 bp) of relict woody plant *M. glyptostroboides* obtained by using Illumina high-throughput sequencing technology. We annotated the genome and performed the repeat sequence analysis. These repeat motifs, identified in the *M. glyptostroboides* cp genome, could be selected for developing markers, population studies, and phylogenetic analysis. Comparison of cp genome structure among land plants suggested that IRA was lost from the *M. glyptostroboides* cp genome. This structural feature found in the *M. glyptostroboides* cp genome was in accord with those of other cp genomes from Cupressophytes, which further supports that there were two independent losses of an IR copy in conifer evolution (i.e., IRA lost in Cupressophytes and IRB lost in Pinaceae). Phylogenetic analysis also suggested that there are two major clades of coniferous species, conifer I (Pinaceae) and conifer II (Cupressophytes). In addition, both MP and ML phylogenetic analyses revealed that *M. glyptostroboides* is a sister species to *C. japonica* and to *T. cryptomerioides*. The data we present here will be great helpful for further investigations of this endemic relict woody plant and also, in conjunction with previously published cp genome sequences, will help to expand our understanding of the evolutionary history of the coniferous cp genomes, especially for the position of *M. glyptostroboides* in plant systematics and evolution.

## Author Contributions

JC designed the experiment, prepared samples and drafted the manuscript. LY, YS, CZ, GL, and WZ performed the experiment. HX contributed to sequence assembling. ZH analyzed and interpreted the data, and drafted the manuscript. JS and TC designed the experiment and drafted the manuscript. All authors contributed to and approved the final manuscript.

## Conflict of Interest Statement

Theauthors declare that the research was conducted in the absence of any commercial or financial relationships that could be construed as a potential conflict of interest.

## Footnotes

1 http://www.ncbi.nlm.nih.gov/genomes/GenomesHome.cgi?taxid=2759&hopt=html

2 http://pgrc.ipk-gatersleben.de/misa/
