# Supplementary material for: The complete chloroplast genome sequence of the relict woody plant Metasequoia glyptostroboides Hu et Cheng
Source: Front Plant Sci. 2015 Jun 16;6:447. doi: 10.3389/fpls.2015.00447 (PMC4468836; doi:10.3389/fpls.2015.00447)
Supplement: Supplementary file 2 [file Table_2.DOCX]

**Table S2.** The codon-anticodon recognition pattern and codon usage for the *M. glyptostroboides* cp genome.

| **Amino acid** | **Codon** | **No.** | **RSCU** | **tRNA** | **Amino acid** | **Codon** | **No.** | **RSCU** | **tRNA** |
| --- | --- | --- | --- | --- | --- | --- | --- | --- | --- |
| Phe | UUU | 977 | 1.4 |  | Tyr | UAU | 733 | 1.62 |  |
| Phe | UUC | 418 | 0.6 | *trnF-GAA* | Tyr | UAC | 172 | 0.38 | *trnY-GUA* |
| Leu | UUA | 901 | 2.02 | *trnL-UAA* | Stop | UAA | 46 | 1.66 |  |
| Leu | UUG | 532 | 1.2 |  | Stop | UAG | 18 | 0.65 |  |
| Leu | CUU | 530 | 1.19 |  | His | CAU | 403 | 1.54 |  |
| Leu | CUC | 178 | 0.4 |  | His | CAC | 121 | 0.46 | *trnH-GUG* |
| Leu | CUA | 385 | 0.86 | *trnL-UAG* | Gln | CAA | 687 | 1.56 | *trnQ-UUG* |
| Leu | CUG | 145 | 0.33 |  | Gln | CAG | 194 | 0.44 |  |
| Ile | AUU | 1028 | 1.48 |  | Asn | AAU | 876 | 1.57 |  |
| Ile | AUC | 348 | 0.5 | *trnI-GAU* | Asn | AAC | 240 | 0.43 | *trnN-GUU* |
| Ile | AUA | 703 | 1.01 | *trnI-CAU* | Lys | AAA | 1171 | 1.51 | *trnK-UUU* |
| Met | AUG | 581 | 1 | *trn(f)M-CAU* | Lys | AAG | 379 | 0.49 |  |
| Val | GUU | 489 | 1.44 |  | Asp | GAU | 839 | 1.61 |  |
| Val | GUC | 160 | 0.47 | *trnV-GAC* | Asp | GAC | 202 | 0.39 | *trnD-GUC* |
| Val | GUA | 496 | 1.46 | *trnV-UAC* | Glu | GAA | 1118 | 1.56 | *trnE-UUC* |
| Val | GUG | 211 | 0.62 |  | Glu | GAG | 315 | 0.44 |  |
| Ser | UCU | 545 | 1.94 |  | Cys | UGU | 203 | 1.5 |  |
| Ser | UCC | 225 | 0.8 | *trnS-GGA* | Cys | UGC | 68 | 0.5 | *trnC-GCA* |
| Ser | UCA | 325 | 1.16 | *trnS-UGA* | Stop | UGA | 19 | 0.69 |  |
| Ser | UCG | 149 | 0.53 |  | Trp | UGG | 436 | 1 | *trnW-CCA* |
| Pro | CCU | 444 | 1.67 |  | Arg | CGU | 343 | 1.45 | *trnR-ACG* |
| Pro | CCC | 202 | 0.76 | *trnP-GGG* | Arg | CGC | 94 | 0.4 |  |
| Pro | CCA | 301 | 1.13 | *trnP-UGG* | Arg | CGA | 307 | 1.29 |  |
| Pro | CCG | 116 | 0.44 |  | Arg | CGG | 78 | 0.33 |  |
| Thr | ACU | 515 | 1.73 |  | Ser | AGU | 342 | 1.22 |  |
| Thr | ACC | 187 | 0.63 |  | Ser | AGC | 97 | 0.35 | *trnS-GCU* |
| Thr | ACA | 361 | 1.21 | *trnT-UGU* | Arg | AGA | 470 | 1.98 | *trnR-UCU* |
| Thr | ACG | 127 | 0.43 |  | Arg | AGG | 132 | 0.56 |  |
| Ala | GCU | 712 | 1.95 |  | Gly | GGU | 580 | 1.49 |  |
| Ala | GCC | 176 | 0.48 |  | Gly | GGC | 142 | 0.36 | *trnG-GCC* |
| Ala | GCA | 432 | 1.19 | *trnA-UGC* | Gly | GGA | 649 | 1.67 | *trnG-UCC* |
| Ala | GCG | 137 | 0.38 |  | Gly | GGG | 186 | 0.48 |  |

RSCU: relative synonymous codon usage.
