# Supplementary material for: The complete chloroplast genome sequence of the relict woody plant Metasequoia glyptostroboides Hu et Cheng
Source: Front Plant Sci. 2015 Jun 16;6:447. doi: 10.3389/fpls.2015.00447 (PMC4468836; doi:10.3389/fpls.2015.00447)
Supplement: Supplementary file 3 [file Table_3.DOCX]

**Table S3.** Repeated sequences in the *M. glyptostroboides* cp genome.

| **Type** | **Number** | **Size(bp)** | **Location** | **Repeat unit** |
| --- | --- | --- | --- | --- |
| **F** | 1 | 44 | IGS(*trnP-GGG*,*rps12*) | TATTTCAGAAAAAATATTTTCCATTCGAAGAACATTATATACAT |
|  | 2 | 32 | IGS(*rpl23*, *ycf2*), IGS(*ndhC*, *trnV-UAC*) | GATGCCTTGATGGTGAAATGGTAGACACGCGA |
|  | 3 | 31 | *trnG-GCC*, *trnG-UCC*(*exon*) | ATGGGCGGGTAGCGGGAATCGAACCCGCATC |
|  | 4 | 33 | *ycf2* | GTAATTCAAAATCGAATTCCAAATAGTATTTAT |
|  | 5 | 30 | IGS(*chlB*, *trnK-UUU*) | ATTGACAATATGCATATTGTTTAAATATAA |
|  | 6 | 34 | IGS(*ndhD*, *trnF-GAA*), IGS(*clpP*, *ccsA*) | CACCCTTTGAAATAACTTGGACGAACTCCCTATT |
|  | 7 | 34 | *psaA*, *psaB* | CGTGGATATTGGCAAGAACTTATTGAATCTATTG |
|  | 8 | 31 | *trnS-UGA*, *trnS-GCU* | TATCGAGGGTTCGAATCCCTCTCTCTCCTTT |
|  | 9 | 31 | *trnK-UUU*(*intron*), IGS(*rbcL*, *accD*) | TTCTTTTTTGATCAGAAGCGGATTTTATCCA |
|  | 10 | 30 | *psaA*, *psaB* | TAATCGCTGGTCATATGTATAGGACCAATT |
|  | 11 | 38 | *rrn23* | TCTCAAGCAGTGGGAGGAGATCTGACCGCCTGCCTGTT |
| **P** | 1 | 278 | IGS(*psbK*, *chlB*), IGS(*trnL-UAA*, *trnT-UGU*) | TGTATTCCCATCCTACACCACAAAAATATCCCTTTCTCTATTTAAATATTATTGAAATCATATTTCCGTTCTATAAGTAGAGTCTACGAAAGGATGAGAAATCAAAAATTCTTTTTGATTTTTTCCAATTTTTCAGAAGAATACGGTTCCACCTGGGACGGAAGGATTCGAACCTTCGCAATAACAGGACCAAAACCTGCTGCCTTACCGCTTGGCCACGCCCCATCGGTGTTGTTTTATTTTAATCAATTAATAGAAATTGATGCAATGTTTTATTG |
|  | 2 | 86 | *trnI-CAU +* IGS(*trnI-CAU*, *ndhB*), IGS(*psbA*, *trnI-CAU*) *+ trnI-CAU* | ATCCATGGCTGAATGGTAAAAGCACCCAACTCATAATTGGGAAGTTGCGGGTTCAATTCCTGCTGGATGCACAATAAACAGTTATT |
|  | 3 | 48 | *rpoC2*, IGS(*ycf1*, *rps12*) | TATACGAACCCCTTTTACTTGCAAAAGTATATCTTGGATTTGTCAATT |
|  | 4 | 35 | IGS(*rps12*, *trnV-GAC*), *ycf3*(*intron*) | CAAAACCGTACATGAGAATTTCACCTCATACGGCT |
|  | 5 | 37 | *rpl16*(*intron*), *rpoC1*(*intron*) | TCAGAACCGGATATGAAAGTTTCTTCTCATCCGGCTC |
|  | 6 | 30 | *trnS-GGA*, *trnS-GCU* | ACGGAAAGAGAGGGATTCGAACCCTCGGTA |
|  | 7 | 30 | *trnS-GGA*, *trnS-UGA* | ACGGAAAGAGAGGGATTCGAACCCTCGGTA |
|  | 8 | 31 | *atpF*(*intron*), IGS(*ndhC*, *trnV-UAC*) | TTAAATGCGGAACCGACGACCTACACAGAAG |
|  | 9 | 33 | IGS(*ndhD*, *trnF-GAA*), IGS(*clpP*, *ccsA*) | TTTTTAGCTTTCAGATGAATTGAAAACTATCTA |
| **T** | 1 | 20 | *rpl16*(*intron*) | TCTTAGTAATTAATATAAAA (×2) |
|  | 2 | 39 | *rps3* | CTTTTTTTTTCTTTTTTTCTTGACCCTGTTTTTTGATAA (×2) |
|  | 3 | 21 | *ycf2* | TTTAATGCTAATTGTTCTTTA (×2) |
|  | 4 | 18 | *ycf2* | ATTCCTCTGATCTTTCAG (×2) |
|  | 5 | 18 | IGS(*ndhB*, *rps7*) | TCAGAATGGAATAGATCT (×2) |
|  | 6 | 21 | IGS(*trnV-GAC*,*rrn16*) | CTAACATTGTTAGTAACATAT (×2) |
|  | 7 | 15 | IGS(*trnV-GAC*,*rrn16*) | AATATAAACAATAAT (×2) |
|  | 8 | 42 | IGS(*trnV-GAC*,*rrn16*) | CCATTTTGAATTTCTCCATTTTCATCTCTATTACTACGAGAA (×2) |
|  | 9 | 25 | IGS(*rrn16*,*trnI-GAU*) | TTTATTAATAAAAGCTTGCTTATTA (×2) |
|  | 10 | 18 | *rpoC2* | CCTTTCCCTTTGAAGTGC (×2) |
|  | 11 | 25 | IGS(*chlB*, *trnK-UUU*) | TTTGGGATTGACAATATGCATATTG (×3) |
|  | 12 | 21 | *ycf1* | ATTTGTTTTGTATCCTTTTTG (×2) |
|  | 13 | 24 | *rps18* | GCTTAGATGTATTTCGAAAAGATG (×9) |
|  | 14 | 29 | IGS(*psbL*, *psbJ*) | ATATGCCTTGAGCATGACTACCTAAGAAA (×2) |
|  | 15 | 16 | IGS(*ycf4*, *psaI*) | TTCTTCTTTTGCCATG (×2) |

F: forward repeats; P: palindromic repeats; T: tandem repeats; IGS: intergenic spacers.
