# Supplementary material for: The complete chloroplast genome sequence of the relict woody plant Metasequoia glyptostroboides Hu et Cheng
Source: Front Plant Sci. 2015 Jun 16;6:447. doi: 10.3389/fpls.2015.00447 (PMC4468836; doi:10.3389/fpls.2015.00447)
Supplement: Supplementary file 4 [file Table_4.DOCX]

**Table S4.** Distribution of SSRs in the *M. glyptostroboides* cp genome.

| **Type** | **Start** | **End** | **Motif** | **Minimum no.of repeats** | **No.of Repeats** | **Location** |
| --- | --- | --- | --- | --- | --- | --- |
| **monomer** | 7,790 | 7,799 | A | 8(8bp) | 10 | IGS(*rps11*, *rpl36*) |
|  | 13,490 | 13,497 | A |  | 8 | *rpl2*(*intron*) |
|  | 28,076 | 28,083 | A |  | 8 | IGS(*trnV-GAC*, *rrn16*) |
|  | 31,576 | 31,583 | A |  | 8 | *trnI-GAU*(*intron*) |
|  | 36,009 | 36,016 | A |  | 8 | IGS(*rrn4.5*, *rrn5*) |
|  | 43,764 | 43,775 | A |  | 12 | *ndhA*(*intron*) |
|  | 49,542 | 49,550 | A |  | 9 | IGS(*trnF-GAA*, *trnL-UAA*) |
|  | 49,809 | 49,822 | A |  | 14 | *trnL-UAA*(*intron*) |
|  | 49,897 | 49,904 | A |  | 8 | *trnL-UAA*(*intron*) |
|  | 51,115 | 51,123 | A |  | 9 | *rps4* |
|  | 51,528 | 51,537 | A |  | 10 | *rps4* |
|  | 53,744 | 53,751 | A |  | 8 | *ycf3*(*intron*) |
|  | 53,955 | 53,964 | A |  | 10 | *ycf3*(*intron*) |
|  | 60,034 | 60,042 | A |  | 9 | IGS(*rps14*, *trnfM-CAU*) |
|  | 64,590 | 64,597 | A |  | 8 | IGS(*psbD*, *trnE-UUC*) |
|  | 64,938 | 64,945 | A |  | 8 | IGS(*psbD*, *trnE-UUC*) |
|  | 66,697 | 66,706 | A |  | 10 | IGS(*trnD-GUC*, *psbM*) |
|  | 67,466 | 67,479 | A |  | 14 | IGS(*trnD-GUC*, *psbM*) |
|  | 67,683 | 67,690 | A |  | 8 | IGS(*psbM*, *petN*) |
|  | 69,576 | 69,585 | A |  | 10 | *rpoB* |
|  | 69,842 | 69,852 | A |  | 11 | *rpoB* |
|  | 73,249 | 73,257 | A |  | 9 | *rpoC1*(*intron*) |
|  | 77,096 | 77,104 | A |  | 9 | *rpoC2* |
|  | 77,398 | 77,405 | A |  | 8 | *rpoC2* |
|  | 78,353 | 78,361 | A |  | 9 | *rpoC2* |
|  | 78,994 | 79,007 | A |  | 14 | *rpoC2* |
|  | 79,227 | 79,234 | A |  | 8 | *rps2* |
|  | 79,587 | 79,597 | A |  | 11 | *rps2* |
|  | 82,661 | 82,668 | A |  | 8 | *atpF*(*intron*) |
|  | 86,524 | 86,533 | A |  | 10 | IGS(*trnG-UCC*, *psaM*) |
|  | 87,808 | 87,815 | A |  | 8 | *psbK* |
|  | 90,448 | 90,458 | A |  | 11 | IGS(*chlB*, *trnK-UUU*) |
|  | 91,430 | 91,437 | A |  | 8 | IGS(*chlB*, *trnK-UUU*) |
|  | 95,886 | 95,893 | A |  | 8 | IGS(*psbA*, *trnI-CAU*) |
|  | 96,820 | 96,827 | A |  | 8 | IGS(*trnH-GUG*, *chlL*) |
|  | 99,582 | 99,590 | A |  | 9 | IGS(*chlN*, *ndhJ*) |
|  | 102,245 | 102,253 | A |  | 9 | IGS(*ndhC*, *trnV-UAC*) |
|  | 102,257 | 102,264 | A |  | 8 | IGS(*ndhC*, *trnV-UAC*) |
|  | 103,018 | 103,025 | A |  | 8 | IGS(*ndhC*, *trnV-UAC*) |
|  | 103,197 | 103,205 | A |  | 9 | IGS(*ndhC*, *trnV-UAC*) |
|  | 108,462 | 108,475 | A |  | 14 | IGS(*rbcL*, *accD*) |
|  | 109,327 | 109,334 | A |  | 8 | *accD* |
|  | 109,760 | 109,767 | A |  | 8 | *accD* |
|  | 111,251 | 111,258 | A |  | 8 | IGS(*accD*, *clpP*) |
|  | 111,296 | 111,304 | A |  | 9 | IGS(*accD*, *clpP*) |
|  | 120,717 | 120,724 | A |  | 8 | *ycf1* |
|  | 121,273 | 121,281 | A |  | 9 | IGS(*ycf1*, *rps12*) |
|  | 121,547 | 121,554 | A |  | 8 | IGS(*ycf1*, *rps12*) |
|  | 122,954 | 122,963 | A |  | 10 | IGS(*rps12*, *rpl20*) |
|  | 123,503 | 123,510 | A |  | 8 | IGS(*rpl20*, *rps18*) |
|  | 124,712 | 124,719 | A |  | 8 | *psaJ* |
|  | 125,321 | 125,328 | A |  | 8 | IGS(*trnP-UGG*, *trnW-CCA*) |
|  | 125,350 | 125,364 | A |  | 15 | IGS(*trnP-UGG*, *trnW-CCA*) |
|  | 126,246 | 126,253 | A |  | 8 | IGS(*petL*, *psbE*) |
|  | 127,780 | 127,791 | A |  | 12 | IGS(*psbJ*, *petA*) |
|  | 127,954 | 127,962 | A |  | 9 | IGS(*psbJ*, *petA*) |
|  | 128,288 | 128,296 | A |  | 9 | IGS(*psbJ*, *petA*) |
|  | 128,354 | 128,363 | A |  | 10 | IGS(*psbJ*, *petA*) |
|  | 6 | 13 | T |  | 8 | IGS(*ycf4*, *psaI*) |
|  | 478 | 486 | T |  | 9 | IGS(*psaI*, *psbB*) |
|  | 1,548 | 1,555 | T |  | 8 | *psbB* |
|  | 3,532 | 3,541 | T |  | 9 | *petB*(*intron*) |
|  | 6,104 | 6,111 | T |  | 8 | IGS(*petD*, *rpoA*) |
|  | 8,756 | 8,764 | T |  | 9 | *rps8* |
|  | 9,056 | 9,066 | T |  | 11 | *rps8* |
|  | 10,578 | 10,589 | T |  | 12 | *rpl16*(*intron*) |
|  | 11,486 | 11,494 | T |  | 9 | *rps3* |
|  | 11,818 | 11,827 | T |  | 10 | IGS(*rps3*, *rpl22*) |
|  | 12,359 | 12,368 | T |  | 10 | IGS(*rpl22*, *rps19*) |
|  | 12,384 | 12,395 | T |  | 12 | *rps19* |
|  | 17,164 | 17,171 | T |  | 8 | *ycf2* |
|  | 21,271 | 21,278 | T |  | 8 | *ycf2* |
|  | 22,649 | 22,656 | T |  | 8 | IGS(*ycf2*, *trnI-CAU*) |
|  | 24,103 | 24,110 | T |  | 8 | *ndhB*(*intron*) |
|  | 24,231 | 24,241 | T |  | 11 | *ndhB*(*intron*) |
|  | 27,174 | 27,181 | T |  | 8 | IGS(*rps12*, *trnV-GAC*) |
|  | 27,234 | 27,241 | T |  | 8 | IGS(*rps12*, *trnV-GAC*) |
|  | 27,813 | 27,822 | T |  | 10 | IGS(*rps12*, *trnV-GAC*) |
|  | 36,337 | 36,344 | T |  | 8 | IGS(*rrn5*, *trnR-ACG*) |
|  | 41,040 | 41,047 | T |  | 8 | IGS(*rpl32*, *rps15*) |
|  | 41,279 | 41,286 | T |  | 8 | IGS(*rpl32*, *rps15*) |
|  | 42,670 | 42,677 | T |  | 8 | *ndhH* |
|  | 43,235 | 43,243 | T |  | 9 | *ndhA* |
|  | 44,046 | 44,055 | T |  | 10 | *ndhA*(*intron*) |
|  | 48,952 | 48,963 | T |  | 12 | IGS(*ndhD*, *trnF-GAA*) |
|  | 51,732 | 51,742 | T |  | 11 | IGS(*rps4*, *trnS-GGA*) |
|  | 53,341 | 53,348 | T |  | 8 | *ycf3*(*intron*) |
|  | 55,055 | 55,063 | T |  | 9 | IGS(*ycf3*, *psaA*) |
|  | 64,018 | 64,026 | T |  | 9 | IGS(*psbD*, *trnE-UUC*) |
|  | 64,416 | 64,424 | T |  | 9 | IGS(*psbD*, *trnE-UUC*) |
|  | 66,717 | 66,731 | T |  | 15 | IGS(*trnD-GUC*, *psbM*) |
|  | 66,829 | 66,836 | T |  | 8 | IGS(*trnD-GUC*, *psbM*) |
|  | 67,452 | 67,463 | T |  | 12 | IGS(*trnD-GUC*, *psbM*) |
|  | 77,615 | 77,622 | T |  | 8 | *rpoC2* |
|  | 81,888 | 81,895 | T |  | 8 | IGS(*atpH*, *atpF*) |
|  | 82,543 | 82,550 | T |  | 8 | *atpF*(*intron*) |
|  | 87,263 | 87,272 | T |  | 10 | IGS(*psbI*, *psbK*) |
|  | 92,682 | 92,689 | T |  | 8 | *trnK-UUU*(*intron*) |
|  | 95,925 | 95,932 | T |  | 8 | IGS(*psbA*, *trnI-CAU*) |
|  | 104,352 | 104,360 | T |  | 9 | IGS(*trnM-CAU*, *atpB*) |
|  | 106,381 | 106,391 | T |  | 11 | IGS(*atpE*, *rbcL*) |
|  | 111,464 | 111,472 | T |  | 9 | *clpP* |
|  | 112,595 | 112,604 | T |  | 10 | IGS(*clpP*, *ccsA*) |
|  | 114,180 | 114,190 | T |  | 11 | IGS(*trnP-GGG*, *ycf1*) |
|  | 115,951 | 115,958 | T |  | 8 | *ycf1* |
|  | 117,046 | 117,053 | T |  | 8 | *ycf1* |
|  | 118,756 | 118,765 | T |  | 10 | *ycf1* |
|  | 122,802 | 122,810 | T |  | 9 | IGS(*rps12*, *rpl20*) |
|  | 123,456 | 123,465 | T |  | 10 | IGS(*rpl20*, *rps18*) |
|  | 124,076 | 124,086 | T |  | 11 | IGS(*rps18*, *rpl33*) |
|  | 125,668 | 125,675 | T |  | 8 | IGS(*petG*, *petL*) |
|  | 126,048 | 126,058 | T |  | 11 | IGS(*petL*, *psbE*) |
|  | 127,978 | 127,986 | T |  | 9 | IGS(*psbJ*, *petA*) |
|  | 128,318 | 128,326 | T |  | 9 | IGS(*psbJ*, *petA*) |
|  | 81,759 | 81,766 | C |  | 8 | IGS(*atpH*, *atpF*) |
|  | 86,193 | 86,200 | C |  | 8 | IGS(*trnG-UCC*, *psaM*) |
|  | 60,574 | 60,582 | G |  | 9 | IGS(*trnG-GCC*, *psbZ*) |
|  | 73,685 | 73,693 | G |  | 9 | *rpoC1*(*intron*) |
|  | 90,955 | 90,963 | G |  | 9 | IGS(*chlB*, *trnK-UUU*) |
|  | 94,480 | 94,489 | G |  | 10 | IGS(*trnK-UUU*, *psbA*) |
| **dimer** | 78,376 | 78,383 | AC | 4(8bp) | 4 | *rpoC2* |
|  | 24,598 | 24,605 | AG |  | 4 | *ndhB* |
|  | 42,060 | 42,067 | AG |  | 4 | *ndhH* |
|  | 69,506 | 69,513 | AG |  | 4 | *rpoB* |
|  | 73,850 | 73,857 | AG |  | 4 | *rpoC1* |
|  | 114,589 | 114,596 | AG |  | 4 | *ycf1* |
|  | 312 | 319 | AT |  | 4 | IGS(*ycf4*, *psaI*) |
|  | 322 | 335 | AT |  | 7 | IGS(*ycf4*, *psaI*) |
|  | 11,580 | 11,589 | AT |  | 5 | *rps3* |
|  | 28,272 | 28,279 | AT |  | 4 | IGS(*trnV-GAC*, *rrn16*) |
|  | 28,341 | 28,348 | AT |  | 4 | IGS(*trnV-GAC*, *rrn16*) |
|  | 52,189 | 52,196 | AT |  | 4 | IGS(*trnS-GGA*, *ycf3*) |
|  | 55,043 | 55,052 | AT |  | 5 | IGS(*ycf3*, *psaA*) |
|  | 65,269 | 65,280 | AT |  | 6 | IGS(*psbD*, *trnE-UUC*) |
|  | 65,309 | 65,316 | AT |  | 4 | IGS(*psbD*, *trnE-UUC*) |
|  | 65,345 | 65,352 | AT |  | 4 | IGS(*psbD*, *trnE-UUC*) |
|  | 76,383 | 76,390 | AT |  | 4 | *rpoC2* |
|  | 76,846 | 76,853 | AT |  | 4 | *rpoC2* |
|  | 100,083 | 100,092 | AT |  | 5 | IGS(*chlN*, *ndhJ*) |
|  | 108,660 | 108,669 | AT |  | 5 | IGS(*rbcL*, *accD*) |
|  | 114,461 | 114,468 | AT |  | 4 | *ycf1* |
|  | 115,965 | 115,972 | AT |  | 4 | *ycf1* |
|  | 122,782 | 122,791 | AT |  | 5 | IGS(*rps12*, *rpl20*) |
|  | 34,731 | 34,738 | CT |  | 4 | *rrn23* |
|  | 61,152 | 61,159 | CT |  | 4 | *trnS-UGA* |
|  | 86,934 | 86,941 | CT |  | 4 | *trnS-GCU* |
|  | 120,998 | 121,005 | CT |  | 4 | *ycf1* |
|  | 127,697 | 127,704 | CT |  | 4 | *psbJ* |
|  | 127,752 | 127,759 | CT |  | 4 | IGS(*psbJ*, *petA*) |
|  | 35,054 | 35,061 | GA |  | 4 | *rrn23* |
|  | 75,125 | 75,132 | GA |  | 4 | *rpoC1* |
|  | 85,330 | 85,337 | GA |  | 4 | *trnG-UCC*(*intron*) |
|  | 90,711 | 90,718 | GA |  | 4 | IGS(*chlB*, *trnK-UUU*) |
|  | 103,254 | 103,261 | GA |  | 4 | IGS(*ndhC*, *trnV-UAC*) |
|  | 7,826 | 7,833 | TA |  | 4 | IGS(*rps11*, *rpl36*) |
|  | 7,902 | 7,909 | TA |  | 4 | IGS(*rps11*, *rpl36*) |
|  | 13,285 | 13,292 | TA |  | 4 | *rpl2*(*intron*) |
|  | 27,081 | 27,088 | TA |  | 4 | IGS(*rps12*, *trnV-GAC*) |
|  | 28,358 | 28,367 | TA |  | 5 | IGS(*trnV-GAC*, *rrn16*) |
|  | 28,850 | 28,857 | TA |  | 4 | IGS(*trnV-GAC*, *rrn16*) |
|  | 36,648 | 36,655 | TA |  | 4 | IGS(*trnR-ACG*, *trnN-GUU*) |
|  | 37,873 | 37,880 | TA |  | 4 | *ndhF* |
|  | 52,258 | 52,265 | TA |  | 4 | IGS(*trnS-GGA*, *ycf3*) |
|  | 61,002 | 61,015 | TA |  | 7 | IGS(*psbZ*, *trnS-UGA*) |
|  | 61,250 | 61,257 | TA |  | 4 | IGS(*trnS-UGA*, *psbC*) |
|  | 69,291 | 69,300 | TA |  | 5 | IGS(*trnC-GCA*, *rpoB*) |
|  | 76,294 | 76,301 | TA |  | 4 | *rpoC2* |
|  | 81,200 | 81,225 | TA |  | 13 | IGS(*atpI*, *atpH*) |
|  | 83,306 | 83,317 | TA |  | 6 | IGS(*atpF*, *atpA*) |
|  | 96,482 | 96,489 | TA |  | 4 | IGS(*trnH-GUG*, *chlL*) |
|  | 99,680 | 99,693 | TA |  | 7 | IGS(*chlN*, *ndhJ*) |
|  | 100,068 | 100,081 | TA |  | 7 | IGS(*chlN*, *ndhJ*) |
|  | 108,644 | 108,659 | TA |  | 8 | IGS(*rbcL*, *accD*) |
|  | 128,304 | 128,317 | TA |  | 7 | IGS(*psbJ*, *petA*) |
| **trimer** | 11,471 | 11,482 | TTC | 4(12bp) | 4 | *rps3* |
|  | 27,186 | 27,197 | ATT |  | 4 | IGS(*rps12*, *trnV-GAC*) |
|  | 116,590 | 116,601 | TTC |  | 4 | *ycf1* |
|  | 116,644 | 116,658 | TTC |  | 5 | *ycf1* |
|  | 120,871 | 120,882 | TTA |  | 4 | *ycf1* |
| **tetramer** | 3,557 | 3,568 | TAAG | 3(12bp) | 3 | *petB* |
|  | 34,693 | 34,704 | AGGT |  | 3 | *rrn23* |
|  | 39,665 | 39,676 | GAAA |  | 3 | *ndhF* |
|  | 41,016 | 41,027 | ATAA |  | 3 | IGS(*rpl32*, *rps15*) |
|  | 80,035 | 80,046 | TCAA |  | 3 | IGS(*rps2*, *atpI*) |
|  | 92,972 | 92,983 | ATGG |  | 3 | *trnK-UUU* |
|  | 106,502 | 106,513 | CATA |  | 3 | IGS(*atpE*, *rbcL*) |
| **pentamer** | 22,492 | 22,511 | TTTAT | 3(15bp) | 4 | IGS(*ycf2*, *trnI-CAU*) |
| **hexamer** | - | - | - | 3(18bp) | 0 | *-* |
