# Supplementary material for: The complete chloroplast genome sequence of the relict woody plant Metasequoia glyptostroboides Hu et Cheng
Source: Front Plant Sci. 2015 Jun 16;6:447. doi: 10.3389/fpls.2015.00447 (PMC4468836; doi:10.3389/fpls.2015.00447)
Supplement: Supplementary file 5 [file Table_5.DOCX]

**Table S5.** GenBank accession numbers of the cp genome sequences used in this study.

| **No.** | **Taxon** | **Order** | **Family** | **GenBank Accession number** |
| --- | --- | --- | --- | --- |
| 1 | *Agathis dammara* | Pinales | Araucariaceae | NC_023119 |
| 2 | *Calocedrus formosana* | Pinales | Cupressaceae | NC_023121 |
| 3 | *Cathaya argyrophylla* | Pinales | Pinaceae | NC_014589 |
| 4 | *Cedrus deodara* | Pinales | Pinaceae | NC_014575 |
| 5 | *Cephalotaxus oliveri* | Pinales | Cephalotaxaceae | NC_021110 |
| 6 | *Cephalotaxus wilsoniana* | Pinales | Cephalotaxaceae | NC_016063 |
| 7 | *Cryptomeria japonica* | Pinales | Cupressaceae | NC_010548 |
| 8 | *Cunninghamia lanceolata* | Pinales | Cupressaceae | NC_021437 |
| 9 | *Cycas revoluta* | Cycadales | Cycadaceae | NC_020319 |
| 10 | *Glycine max* | Fabales | Fabaceae | NC_007942 |
| 11 | *Ginkgo biloba* | Ginkgoales | Ginkgoaceae | NC_016986 |
| 12 | *Juniperus bermudiana* | Pinales | Cupressaceae | NC_024021 |
| 13 | *Juniperus monosperma* | Pinales | Cupressaceae | NC_024022 |
| 14 | *Juniperus scopulorum* | Pinales | Cupressaceae | NC_024023 |
| 15 | *Juniperus virginiana* | Pinales | Cupressaceae | NC_024024 |
| 16 | *Keteleeria davidiana* | Pinales | Pinaceae | NC_011930 |
| 17 | *Larix decidua* | Pinales | Pinaceae | NC_016058 |
| 18 | *Nageia nagi* | Pinales | Podocarpaceae | NC_023120 |
| 19 | *Nymphaea alba* | Nymphaeales | Nymphaeaceae | NC_006050 |
| 20 | *Oryza australiensis* | Poales | Poaceae | NC_024608 |
| 21 | *Picea abies* | Pinales | Pinaceae | NC_021456 |
| 22 | *Picea morrisonicola* | Pinales | Pinaceae | NC_016069 |
| 23 | *Pinus koraiensis* | Pinales | Pinaceae | NC_004677 |
| 24 | *Pinus massoniana* | Pinales | Pinaceae | NC_021439 |
| 25 | *Pinus taeda* | Pinales | Pinaceae | NC_021440 |
| 26 | *Pinus thunbergii* | Pinales | Pinaceae | NC_001631 |
| 27 | *Podocarpus lambertii* | Pinales | Podocarpaceae | NC_023805 |
| 28 | *Podocarpus totara* | Pinales | Podocarpaceae | NC_020361 |
| 29 | *Pseudotsuga sinensis var. wilsoniana* | Pinales | Pinaceae | NC_016064 |
| 30 | *Taiwania cryptomerioides* | Pinales | Cupressaceae | NC_016065 |
| 31 | *Taiwania flousiana* | Pinales | Cupressaceae | NC_021441 |
| 32 | *Taxus mairei* | Pinales | Taxaceae | KJ123824 |
