# Supplementary material for: The complete chloroplast genome sequence of the relict woody plant Metasequoia glyptostroboides Hu et Cheng
Source: Front Plant Sci. 2015 Jun 16;6:447. doi: 10.3389/fpls.2015.00447 (PMC4468836; doi:10.3389/fpls.2015.00447)
Supplement: Supplementary file 6 [file Table_6.DOCX]

**Table S6.** Average pairwise sequence distances of protein-coding genes from the cp genomes of 28 conifers.

| **Order** | **Gene** | **D** | **SE** | **Average length (bp)** | **Range of length (bp)** |
| --- | --- | --- | --- | --- | --- |
| 1 | *ycf1* | 0.59912 | 0.01307 | 6683 | 5004-8397 |
| 2 | *clpP* | 0.45934 | 0.02343 | 679 | 573-1392 |
| 3 | *ycf2* | 0.43459 | 0.00712 | 6750 | 6165-7446 |
| 4 | *infA* | 0.33422 | 0.02951 | 249 | 228-270 |
| 5 | *accD* | 0.30536 | 0.01564 | 1856 | 963-3171 |
| 6 | *rpl22* | 0.24442 | 0.01971 | 417 | 360-477 |
| 7 | *psaM* | 0.23078 | 0.03448 | 93 | 87-102 |
| 8 | *psaI* | 0.19530 | 0.03523 | 117 | 105-171 |
| 9 | *rpl32* | 0.19433 | 0.02751 | 223 | 120-351 |
| 10 | *psbM* | 0.18847 | 0.03107 | 110 | 99-114 |
| 11 | *rps3* | 0.18840 | 0.01160 | 680 | 654-744 |
| 12 | *matK* | 0.18474 | 0.00724 | 1534 | 1509-1560 |
| 13 | *rpl23* | 0.18092 | 0.01878 | 285 | 273-306 |
| 14 | *rps18* | 0.18025 | 0.02136 | 338 | 243-450 |
| 15 | *petL* | 0.17522 | 0.03164 | 125 | 96-189 |
| 16 | *rps15* | 0.17496 | 0.01827 | 264 | 261-267 |
| 17 | *rps4* | 0.16890 | 0.01223 | 627 | 597-690 |
| 18 | *rpoC1* | 0.16682 | 0.00635 | 2134 | 2028-2448 |
| 19 | *rps2* | 0.16628 | 0.01031 | 708 | 696-759 |
| 20 | *rpoA* | 0.16599 | 0.00962 | 1003 | 972-1029 |
| 21 | *rps11* | 0.16028 | 0.01375 | 406 | 378-462 |
| 22 | *psaJ* | 0.15899 | 0.02308 | 133 | 123-153 |
| 23 | *rps19* | 0.15515 | 0.01628 | 303 | 279-390 |
| 24 | *rpoC2* | 0.15449 | 0.00525 | 3533 | 3021-3729 |
| 25 | *rpoB* | 0.15375 | 0.00479 | 3263 | 3225-3336 |
| 26 | *atpE* | 0.15056 | 0.01288 | 411 | 405-429 |
| 27 | *rpl36* | 0.14958 | 0.02649 | 114 | 114 |
| 28 | *psbJ* | 0.14071 | 0.02349 | 122 | 114-123 |
| 29 | *rpl2* | 0.13985 | 0.00908 | 831 | 825-837 |
| 30 | *rps7* | 0.13443 | 0.01213 | 480 | 396-840 |
| 31 | *rps8* | 0.13428 | 0.01173 | 399 | 399 |
| 32 | *ccsA* | 0.13208 | 0.00804 | 954 | 909-963 |
| 33 | *atpF* | 0.13156 | 0.00991 | 555 | 546-564 |
| 34 | *rpl20* | 0.13045 | 0.01238 | 357 | 336-390 |
| 35 | *cemA* | 0.12820 | 0.00801 | 788 | 783-834 |
| 36 | *rps14* | 0.12572 | 0.01454 | 302 | 300-303 |
| 37 | *psbH* | 0.12561 | 0.01490 | 228 | 225-228 |
| 38 | *psbK* | 0.12546 | 0.01761 | 183 | 180-186 |
| 39 | *rpl33* | 0.12011 | 0.01540 | 203 | 195-207 |
| 40 | *ndhK* | 0.10599 | 0.00869 | 790 | 765-807 |
| 41 | *chlN* | 0.10287 | 0.00568 | 1390 | 1368-1437 |
| 42 | *ycf4* | 0.10249 | 0.00843 | 554 | 546-555 |
| 43 | *chlB* | 0.10052 | 0.00466 | 1541 | 1533-1551 |
| 44 | *ndhJ* | 0.09977 | 0.00845 | 492 | 477-522 |
| 45 | *rpl16* | 0.09856 | 0.01037 | 418 | 405-450 |
| 46 | *petA* | 0.09520 | 0.00594 | 968 | 960-1008 |
| 47 | *rpl14* | 0.09519 | 0.01065 | 369 | 369 |
| 48 | *ndhF* | 0.09516 | 0.00420 | 2219 | 2208-2238 |
| 49 | *atpB* | 0.09400 | 0.00526 | 1480 | 1464-1533 |
| 50 | *atpA* | 0.09300 | 0.00489 | 1520 | 1485-1527 |
| 51 | *rps12* | 0.09028 | 0.01178 | 373 | 369-405 |
| 52 | *ndhB* | 0.09005 | 0.00500 | 1487 | 1479-1539 |
| 53 | *atpI* | 0.08764 | 0.00678 | 748 | 747-750 |
| 54 | *ndhC* | 0.08667 | 0.00944 | 363 | 363 |
| 55 | *psbZ* | 0.08326 | 0.01383 | 201 | 189-300 |
| 56 | *psbI* | 0.08032 | 0.01618 | 111 | 111-114 |
| 57 | *psbL* | 0.08025 | 0.01620 | 118 | 117-126 |
| 58 | *psbT* | 0.07802 | 0.01727 | 108 | 108 |
| 59 | *chlL* | 0.07799 | 0.00610 | 874 | 864-879 |
| 60 | *ndhG* | 0.07721 | 0.00744 | 543 | 534-546 |
| 61 | *ndhD* | 0.07542 | 0.00401 | 1508 | 1500-1524 |
| 62 | *petG* | 0.07299 | 0.01642 | 114 | 114 |
| 63 | *petB* | 0.06896 | 0.00606 | 648 | 648 |
| 64 | *rbcL* | 0.06826 | 0.00424 | 1428 | 1428 |
| 65 | *psaA* | 0.06751 | 0.00326 | 2254 | 2250-2262 |
| 66 | *petN* | 0.06741 | 0.02003 | 90 | 90 |
| 67 | *psaB* | 0.06568 | 0.00337 | 2205 | 2205 |
| 68 | *psbB* | 0.06507 | 0.00416 | 1527 | 1527 |
| 69 | *psbC* | 0.06455 | 0.00444 | 1422 | 1422 |
| 70 | *ndhA* | 0.06399 | 0.00468 | 1106 | 1098-1107 |
| 71 | *atpH* | 0.06383 | 0.01028 | 246 | 246 |
| 72 | *ycf3* | 0.06054 | 0.00677 | 512 | 507-525 |
| 73 | *psbN* | 0.05935 | 0.01312 | 132 | 132 |
| 74 | *petD* | 0.05872 | 0.00725 | 517 | 483-675 |
| 75 | *ndhI* | 0.05794 | 0.00648 | 498 | 483-552 |
| 76 | *psbF* | 0.05748 | 0.01375 | 120 | 120 |
| 77 | *ndhE* | 0.05625 | 0.00831 | 303 | 303 |
| 78 | *psbE* | 0.05364 | 0.00837 | 255 | 252-258 |
| 79 | *ndhH* | 0.05351 | 0.00421 | 1180 | 1176-1182 |
| 80 | *psbA* | 0.05067 | 0.00441 | 1062 | 1062 |
| 81 | *psbD* | 0.04648 | 0.00421 | 1062 | 1062-1068 |
| 82 | *psaC* | 0.04224 | 0.00802 | 246 | 246 |

D and SE indicate average sequence distances and standard errors, respectively.
