# Supplementary material for: The complete chloroplast genome sequence of the relict woody plant Metasequoia glyptostroboides Hu et Cheng
Source: Front Plant Sci. 2015 Jun 16;6:447. doi: 10.3389/fpls.2015.00447 (PMC4468836; doi:10.3389/fpls.2015.00447)
Supplement: Supplementary file 7 [file Table_7.DOCX]

**Table S7.** The index of substitution saturation **(**Iss) values of 64 protein-coding genes common to 30 species.

| **Order** | **Gene** | **Iss** | **Iss.c** | |
| --- | --- | --- | --- | --- |
|  |  |  | For a symmetrical tree | For an asymmetrical tree |
| 1 | *psaC* | 0.0534 | 0.6565 | 0.3597 |
| 2 | *psbA* | 0.0629 | 0.7563 | 0.4731 |
| 3 | *psbF* | 0.0680 | 0.6378 | 0.4262 |
| 4 | *psbD* | 0.0711 | 0.7566 | 0.4738 |
| 5 | *atpH* | 0.0728 | 0.6565 | 0.3597 |
| 6 | *psbN* | 0.0750 | 0.6368 | 0.4069 |
| 7 | *psbB* | 0.0770 | 0.7752 | 0.5122 |
| 8 | *psaB* | 0.0776 | 0.7897 | 0.5422 |
| 9 | *rbcL* | 0.0795 | 0.7722 | 0.5054 |
| 10 | *petB* | 0.0797 | 0.7242 | 0.4175 |
| 11 | *petG* | 0.0818 | 0.6388 | 0.4384 |
| 12 | *psbC* | 0.0841 | 0.7720 | 0.5050 |
| 13 | *petN* | 0.0860 | 0.6504 | 0.5129 |
| 14 | *psaA* | 0.0924 | 0.7907 | 0.5438 |
| 15 | *psbT* | 0.0926 | 0.6405 | 0.4526 |
| 16 | *psbZ* | 0.1005 | 0.6439 | 0.3658 |
| 17 | *psbE* | 0.1083 | 0.6593 | 0.3603 |
| 18 | *rpl14* | 0.1130 | 0.6827 | 0.3759 |
| 19 | *atpI* | 0.1133 | 0.7344 | 0.4326 |
| 20 | *ycf4* | 0.1265 | 0.7127 | 0.4053 |
| 21 | *atpA* | 0.1329 | 0.7753 | 0.5126 |
| 22 | *psbH* | 0.1426 | 0.6524 | 0.3598 |
| 23 | *chlB* | 0.1474 | 0.7757 | 0.5140 |
| 24 | *rps14* | 0.1485 | 0.6693 | 0.3650 |
| 25 | *rpl36* | 0.1533 | 0.6388 | 0.4384 |
| 26 | *rps8* | 0.1566 | 0.6882 | 0.3815 |
| 27 | *ycf3* | 0.1586 | 0.7088 | 0.4026 |
| 28 | *psbI* | 0.1621 | 0.6388 | 0.4384 |
| 29 | *psbJ* | 0.1777 | 0.6374 | 0.4208 |
| 30 | *rpl2* | 0.1823 | 0.7421 | 0.4459 |
| 31 | *atpF* | 0.2013 | 0.7139 | 0.4063 |
| 32 | *rpl33* | 0.2106 | 0.6477 | 0.3618 |
| 33 | *psbK* | 0.2132 | 0.6433 | 0.3667 |
| 34 | *atpB* | 0.2251 | 0.7756 | 0.5136 |
| 35 | *chlN* | 0.2252 | 0.7727 | 0.5063 |
| 36 | *rps15* | 0.2268 | 0.6613 | 0.3609 |
| 37 | *petA* | 0.2336 | 0.7536 | 0.4677 |
| 38 | *chlL* | 0.2601 | 0.7485 | 0.4579 |
| 39 | *atpE* | 0.2791 | 0.6934 | 0.3872 |
| 40 | *rpoB* | 0.3091 | 0.8009 | 0.5654 |
| 41 | *cemA* | 0.3244 | 0.7421 | 0.4459 |
| 42 | *matK* | 0.3248 | 0.7767 | 0.5169 |
| 43 | *rpl16* | 0.3288 | 0.6973 | 0.3917 |
| 44 | *psbL* | 0.3298 | 0.6369 | 0.4112 |
| 45 | *ccsA* | 0.3414 | 0.7543 | 0.4691 |
| 46 | *psbM* | 0.3451 | 0.6388 | 0.4384 |
| 47 | *rpoA* | 0.3452 | 0.7565 | 0.4735 |
| 48 | *rpl20** | 0.4236 | 0.6871 | 0.3804 |
| 49 | *rps2** | 0.5464 | 0.7405 | 0.4430 |
| 50 | *psaJ** | 0.5508 | 0.6380 | 0.3845 |
| 51 | *infA** | 0.5627 | 0.6627 | 0.3614 |
| 52 | *rps4** | 0.5838 | 0.7325 | 0.4295 |
| 53 | *rps3** | 0.6100 | 0.7383 | 0.4391 |
| 54 | *rpoC1** | 0.7228 | 0.7963 | 0.5530 |
| 55 | *rps11** | 0.7540 | 0.7079 | 0.4021 |
| 56 | *rpoC2** | 0.7776 | 0.8024 | 0.5775 |
| 57 | *petD** | 0.8054 | 0.7304 | 0.4263 |
| 58 | *rps19** | 0.9120 | 0.6903 | 0.3838 |
| 59 | *rpl22** | 0.9750 | 0.7139 | 0.4063 |
| 60 | *psaI** | 1.0859 | 0.6405 | 0.3729 |
| 61 | *rps18** | 1.1383 | 0.7015 | 0.3968 |
| 62 | *petL** | 1.1850 | 0.6439 | 0.3658 |
| 63 | *rpl32** | 1.5481 | 0.6919 | 0.3855 |
| 64 | *accD** | 1.7502 | 0.8014 | 0.5697 |

Iss: an entropy-based index of substitution saturation proposed by Xia et al (2003). Iss.c: the critical Iss value. One asterisk after a gene indicate that the Iss value of that gene is greater than the Iss.c value.
